# Capillary contact points determine beta cell polarity, control secretion and are disrupted in the *db*/*db* mouse model of diabetes

**DOI:** 10.1007/s00125-024-06180-x

**Published:** 2024-05-30

**Authors:** Dillon Jevon, Louise Cottle, Nicole Hallahan, Richard Harwood, Jaswinder S. Samra, Anthony J. Gill, Thomas Loudovaris, Helen E. Thomas, Peter Thorn

**Affiliations:** 1 Charles Perkins Centre, School of Medical Sciences, University of Sydney, Camperdown, NSW Australia; 2 Charles Perkins Centre, Sydney Microscopy and Microanalysis, University of Sydney, Camperdown, NSW Australia; 3 The University of Sydney Northern Clinical School, Sydney, NSW Australia; 4 Upper Gastrointestinal Surgical Unit, Royal North Shore Hospital, St Leonards, NSW Australia; 5 Department of Anatomical Pathology, Royal North Shore Hospital, St Leonards, NSW Australia; 6 Cancer Diagnosis and Pathology Research Group, Kolling Institute of Medical Research, St Leonards, NSW Australia; 7 St Vincent’s Institute, Fitzroy, VIC Australia; 8 Department of Medicine, St Vincent’s Hospital, University of Melbourne, Fitzroy, VIC Australia

**Keywords:** Beta cell, Diabetes, Human, Insulin, Islet, Polarity

## Abstract

**Aims/hypothesis:**

Almost all beta cells contact one capillary and insulin granule fusion is targeted to this region. However, there are reports of beta cells contacting more than one capillary. We therefore set out to determine the proportion of beta cells with multiple contacts and the impact of this on cell structure and function.

**Methods:**

We used pancreatic slices in mice and humans to better maintain cell and islet structure than in isolated islets. Cell structure was assayed using immunofluorescence and 3D confocal microscopy. Live-cell two-photon microscopy was used to map granule fusion events in response to glucose stimulation.

**Results:**

We found that 36% and 22% of beta cells in islets from mice and humans, respectively, have separate contact with two capillaries. These contacts establish a distinct form of cell polarity with multiple basal regions. Both capillary contact points are enriched in presynaptic scaffold proteins, and both are a target for insulin granule fusion. Cells with two capillary contact points have a greater capillary contact area and secrete more, with analysis showing that, independent of the number of contact points, increased contact area is correlated with increased granule fusion. Using *db*/*db* mice as a model for type 2 diabetes, we observed changes in islet capillary organisation that significantly reduced total islet capillary surface area, and reduced area of capillary contact in single beta cells.

**Conclusions/interpretation:**

Beta cells that contact two capillaries are a significant subpopulation of beta cells within the islet. They have a distinct form of cell polarity and both contact points are specialised for secretion. The larger capillary contact area of cells with two contact points is correlated with increased secretion. In the *db*/*db* mouse, changes in capillary structure impact beta cell capillary contact, implying that this is a new factor contributing to disease progression.

**Graphical Abstract:**

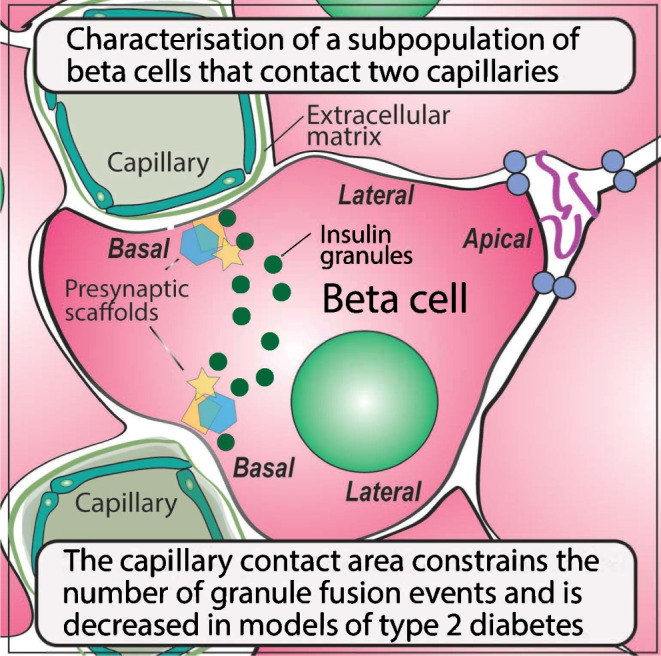

**Supplementary Information:**

The online version contains peer-reviewed but unedited supplementary material available at 10.1007/s00125-024-06180-x.


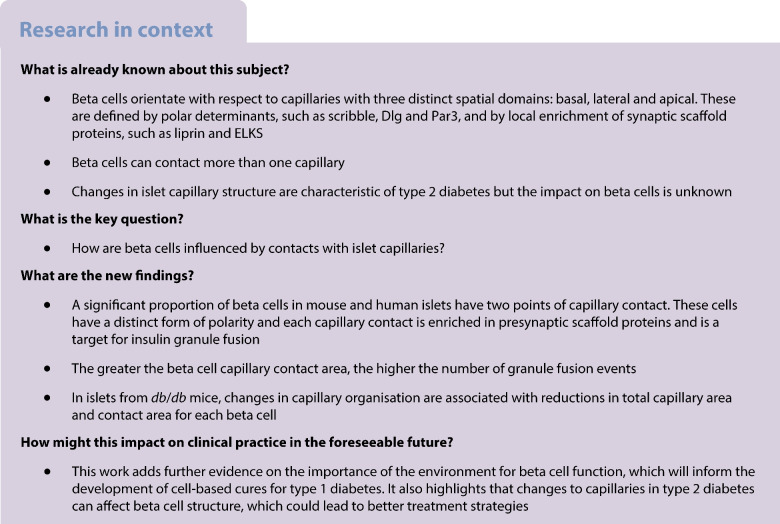


## Introduction

Indirect data suggest beta cells might be polarised, with evidence of polar distribution of viral particles [1], insulin granules [2] and the Golgi apparatus [3]. More recently, a regulator of cell polarity, liver kinase B1 (LKB1), was shown to control beta cell structure [4], and direct evidence shows segregated distributions of polarity determinant complexes indicating a basal region at the capillary interface, a lateral region where endocrine cells contact each other and an apical region that defines luminal spaces into which each beta cell projects a primary cilium [5].

This polar organisation of beta cells is paralleled by the spatial organisation of functional domains. The GLUT2 transporter is enriched laterally [6], ribosomes [7] and primary cilia [5, 8] are apical and presynaptic scaffold proteins are enriched basally at the interface with the capillary [5, 9, 10]. These presynaptic scaffold proteins, such as ELKS, liprin and Rab interacting molecule 2 (RIM2), are important in controlling insulin secretion [11–14] and their enrichment at the capillary interface spatially coincides with targeting of insulin granule fusion to the capillaries [10]. The mechanisms that orientate and organise beta cell structure are unknown. The contact point of the beta cell with the capillaries is the only region where beta cells interact with the extracellular matrix (ECM) [9, 15, 16]. This locally activates integrins [9] and, by analogy with the mechanisms that polarise epithelial cells, could trigger sorting mechanisms to define this as the basal domain [17].

The environment of the islet is complex, with a tortuous and tightly packed capillary bed [18]. Bonner-Weir [2] and our laboratory [5] have reported that, as a result, beta cells can have multiple points of contact with the islet capillaries, with previous speculation that each contact point might have different functions [2]. An obvious question then is, what does cell polarity look like in these beta cells and what consequence might it have for beta cell structure and function?

Furthermore, in type 2 diabetes the islet capillary bed and endothelial cells undergo changes [19–23]. It would seem natural that these changes might have a consequence for the interaction of beta cells with the capillaries and potentially an impact on beta cell function.

## Methods

### Animal husbandry

Mice were housed at the Charles Perkins Centre in a pathogen-free environment, at 22°C with 12 h light cycles and a chow diet. Mice (8–12 weeks old) were humanely killed according to animal ethics procedures (University of Sydney Ethics Committee, no. 20191642 and no. 2023/2300).

Male LepR^*db*/*db*^ mice were genotyped and phenotyped (B6.BKS(D)-*Lepr*^db^/J [The Jackson Laboratory, USA; https://www.jax.org/strain/000697]). Diabetes severity was classified based on AUC analysis of IPGTTs, beginning at stage 1 with compensation (AUC <1600 mmol/l × min) and ending at stage 4 with severe diabetes (AUC >2800 mmol/l × min) (see [24] for details). All LepR^*db*/*db*^ mice in this work exhibited stage 4 type 2 diabetes at 12 weeks.

### Human pancreatic tissue

Human pancreatic tissue was sourced from pancreatic tumour resections or cadaveric donors. Tumour resections were performed at the Royal North Shore Hospital and tissue was collected with patient consent, approved by the Northern Sydney Local Health District Human Research Ethics Committee. Fixed pancreatic sections were from the JDRF Network for Pancreatic Organ donors with Diabetes (nPOD) tissue bank, approved by the Human Research Ethics Committee at the University of Sydney. Cadaveric donor tissue and islets were sourced from St Vincent’s Institute. Informed consent was acquired, and the study was approved by the Human Research Ethics Committee at the University of Sydney. Information on pancreatic tissue donors and islet donors/preparations is listed in electronic supplementary material (ESM) Table 1.

### Pancreatic slicing

The mouse pancreas was inflated by injecting 2 ml of 37°C 1% agarose (Ultrapure Low Melting Point, Invitrogen, ThermoFisher, Australia). The pancreas was excised, trimmed and placed into 15 mm × 15 mm × 5 mm tissue moulds (Tissue-Tek Cryomold, Sakura, Emgrid, Australia), and tissue blocks cast by pipetting 1 ml of 37°C 2% agarose and solidifying on ice.

Human pancreatic tissue was fixed using 4% paraformaldehyde (Sigma-Aldrich, Merck, Australia) at 4°C for 2.5 h. Tissue was washed before storage at 4°C in PBS supplemented with 0.01% sodium azide. Fixed tissue was mounted in 1.5% low-melting-point agarose as above.

Tissue blocks (mouse or human) were sectioned (150–200-µm-thick slices) on a vibratome (Leica VT1000s, Leica, Australia). For fixed-cell imaging experiments, mouse slices were then fixed in 0.5 ml of 4% paraformaldehyde and washed three times in PBS before being stained.

For live-cell assays, mouse slices were cultured in RPMI-1640 culture medium (Sigma-Aldrich), 10.7 mmol/l glucose, supplemented with 10% FBS (Gibco, ThermoFisher, Australia), 100 U/ml penicillin/0.1 mg/ml streptomycin (Invitrogen), 1 μM dexamethasone and 0.01% soybean trypsin inhibitor, overnight in an incubator (37°C, 95% air/5% CO_2_), the medium being replaced 2 h after slicing.

### Immunolabelling

Fixed pancreatic slices were incubated in blocking buffer (3% BSA, 3% donkey serum, 0.3% Triton X-100) for 3 h at room temperature followed by primary antibody at 4°C overnight. Slices were washed three times in PBS and secondary antibodies in blocking buffer were added for 4 h at 4°C. After washing three times in PBS, tissues were mounted on slides with Prolong Diamond anti-fade (Invitrogen). Slides were stored at 4°C and imaged within 5 days.

### Antibodies

Primary antibodies are shown in Table 1. Secondary antibodies were goat anti-guinea pig Alexa Fluor 488, goat anti-mouse Alexa Fluor 546, goat anti-rabbit Alexa Fluor 594 and goat anti-rat Alexa Fluor 647 (Invitrogen), diluted 1:200 in blocking buffer. DAPI (Sigma-Aldrich, 100 ng/ml final concentration) was added with the secondary antibodies.

**Table 1:** Primary antibodies

Target	Species	Dilution	Identifier
Insulin	Guinea pig	Stock	Agilent, A0564; RRID:AB_10013624
Dlg	Mouse	1:200	BD Biosciences, 610874; RRID:AB_398191
E-cadherin	Mouse	1:200	BD Biosciences, 610181; RRID:AB_397580
ELKS	Mouse	1:200	Abcam, Ab50312; RRID:AB_869944
Laminin beta-1	Rat	1:200	Thermo Fisher, MA5-14657; RRID:AB_10981503
Liprin α1	Rabbit	1:200	Proteintech, 14175-2-AP; RRID:AB_2171592
Par3	Rabbit	1:200	Millipore, 07-330; RRID:AB_2101325
pFAK (Y397)	Rabbit	1:100	Cell Signaling, 8556; RRID:AB_10891442

### Confocal image acquisition

Images were acquired on a Leica SP8 confocal microscope with a ×40 or ×63 oil immersion lens, and excited with tuneable white light and an ultraviolet laser. Z stacks were acquired at 255 nm intervals (mouse islets) or 336 nm intervals (human islets) over a depth of around 40 µm.

### 3D cell polarity analysis

Cells with two capillary contacts were located in the confocal Z stacks. To quantify protein localisation at the basal, lateral and apical domains, 5-pixel-wide linescans were drawn along the cell membrane of each domain (Fiji [25]). Average fluorescence intensity of the line was measured on multiple Z planes, and the average intensity of measurements over all planes was calculated for each fluorescence channel of interest at every domain. For each channel, the sum of the fluorescence intensities in all these regions was calculated. The fluorescence intensity calculated for each domain was then expressed as a proportion (%) of this total as shown in the graphs in Figs 2, 3, 4 and 5.

### 3D image segmentation

Confocal fluorescence data were segmented with Avizo (ThermoFisher, Australia). Laminin staining identified capillaries and the islet capsule, membrane staining defined cell boundaries and insulin staining defined beta cells. A 7-pixel-wide brush was used to draw segmentation boundaries for cells, capillaries and capsules, and data were interpolated between two and seven optical slices.

### Live-cell image acquisition

Slice medium was replaced with Krebs Ringer bicarbonate buffer (KRBH), 2.8 mmol/l glucose, for a pre-basal period of 1 h prior to imaging. Then, 1 ml of KRBH (2.8 mmol/l glucose, 1 mmol/l sulforhodamine B [SRB] dye) was added to the microscope imaging chamber and pre-heated to 37°C. Live-cell imaging was done on a homemade multi-photon microscope with an Olympus IX71 body and a ×60 Olympus PlanApo N oil immersion objective lens. Single slices were imaged in a heat conductive, open brass chamber which held 25 mm round coverslips on a heated platform (37°C). Slices were held stationary in solution by a slice anchor (Harvard Bioscience, MA, USA).

Fluorescence was excited at 810 nm (Chameleon, Coherent Scientific, Australia) and emission captured at 6 frames per second (Photo-sensor module H7422PA-40, Hamamatsu Photonics, Hamamatsu, Japan) and visualised with Scanimage (MBF Bioscience, VT, USA) software. The SRB (peak emission 577 nm) signal was collected at >590 nm.

### Exocytosis assay

Data were analysed via Metamorph software (Molecular Devices, USA). Frames were manually screened to identify granule fusion events, which appear as sudden, circular flashes ~1 μm in diameter and decay over 1–3 s (3–9 frames) (Fig. 5). Once a granule fusion event had been visualised, 0.7-μm-diameter regions of interest (ROIs) were placed over the location, and granule fusion was confirmed by kymograph examination and analysed using Microsoft Excel (Microsoft 365, Microsoft, USA).

### Electron microscopy

**Fig. 1:**
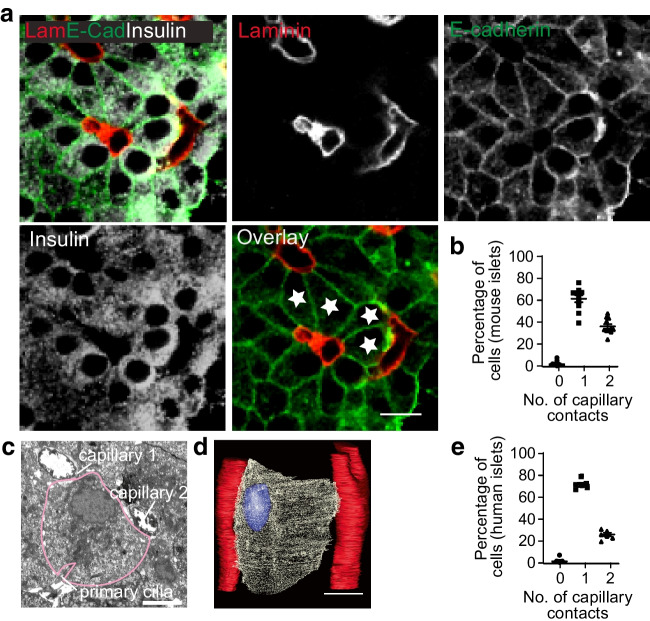
Beta cells contacting two capillaries are a significant proportion of the total beta cell population. (**a**) Laminin (Lam) immunostaining identifies islet capillaries, insulin the beta cells and E-cadherin (E-cad) the cell outlines. Many beta cells contact two capillaries, for example, those identified with a star in the overlay image. (**b**) In mouse islets, within a pancreatic slice, serial confocal sections identify that 36% of cells contact two capillaries (*n*=11 islets, 858 beta cells, 6 mice). (**c**) An example electron micrograph of a single beta cell contacting two capillaries. Scale bar, 5 µm. (**d**) Reconstruction of serial electron micrograph sections showing a single beta cell and two capillary contacts. Scale bar, 5 µm. (**e**) In human islets, within slices, 22% of beta cells contact two capillaries (*n*=5 islets from 2 donors, 262 beta cells)

Serial block face imaging (Fig. 1) was performed as previously described [5].

### Statistics

Data are expressed as scatter plots. Statistical analyses used GraphPad Prism 10 (Dotmatics, USA) with two-sided Student’s *t* tests, one-way ANOVA, and Mann–Whitney and Spearman’s tests where appropriate, with levels of significance as indicated. Randomisation and blinding were not carried out and there were no inclusion or exclusion criteria for the samples or data. To obtain an unbiased dataset from thousands of optical sections we selected data from across at least three individuals and from multiple islets per animal, as indicated in the text.

## Results

Here we use the pancreatic slice technique, for superior preservation of islet structure compared with isolated islets or paraffin sections [5, 26–28], to determine the precise relationship of beta cells with the islet capillaries.

With fixed-cell immunofluorescence in mouse and human islets, we collected hundreds of serial optical sections through the islets. Immunostaining of laminin marked the capillaries. Laminin is an ECM protein exclusively secreted by capillary wall cells [15]. We counter immunostained for insulin to identify beta cells and for epithelial cadherin (E-cadherin), a cell junction protein, to outline the cells (Fig. 1a). We assessed, in three-dimensions for each cell, the number of capillary contacts, showing most mouse beta cells have one capillary contact but ~30% have two (Fig. 1b). Multiple points of capillary contact were also seen in serial electron micrograph sections (Fig. 1c,d). Furthermore, analysis of human islets also showed a significant proportion of beta cells with two points of capillary contact (Fig. 1e).

### Structural organisation of beta cells with multiple capillary contacts

The canonical organisation of polarised epithelia is one basal pole, a lateral domain and one apical pole [17]. For beta cells with one point of capillary contact there is a similar organisation [5, 17]. Here we set out to determine the effect of contact with two capillaries on beta cell polarity.

**Fig. 2:**
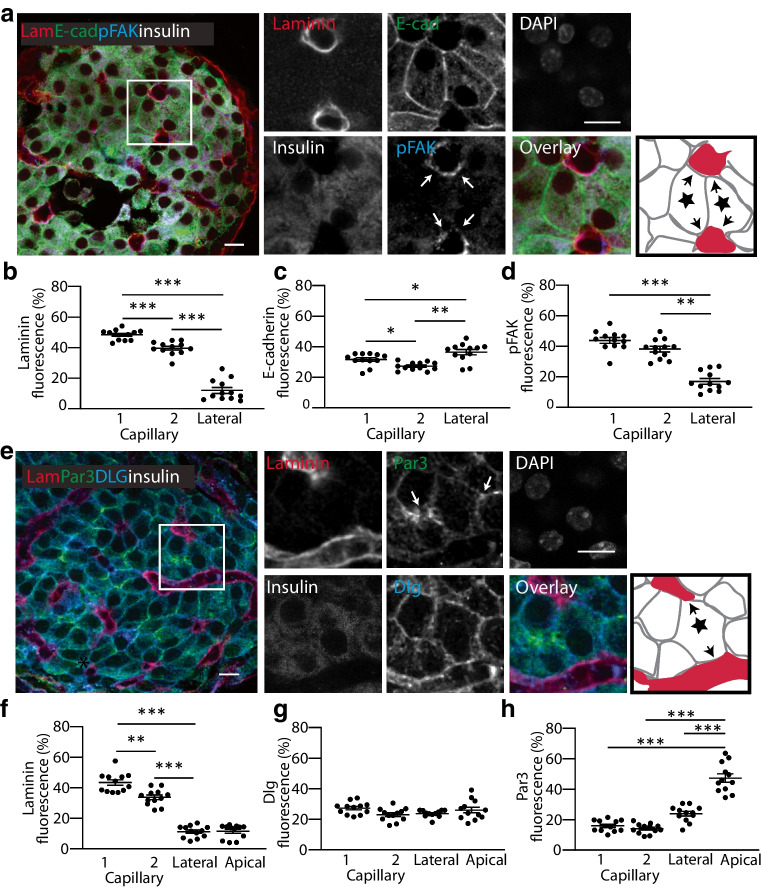
In mouse islets, regional enrichment of membrane domains and segregated distribution of polarity determinants, Dlg and Par3, in cells that contact two capillaries. (**a**) Example image of the combined immunostaining of laminin (Lam), E-cadherin (E-cad) and pFAK. Enlarged images within the square ROI are shown, with arrows highlighting pFAK staining. The cartoon identifies, with stars, two beta cells with two capillary contacts (shown with arrows). (**b**) Laminin is significantly enriched at both capillary interfaces compared with the lateral surface. (**c**) E-cadherin is significantly enriched in the lateral domain compared with both capillary interfaces. (**d**) pFAK is significantly enriched at both capillary interfaces compared with the lateral surface. (**e**) Combined staining of laminin, Dlg and Par3. Enlarged images within the square ROI are shown, with arrows highlighting Par3 staining. The cartoon identifies, with a star, a beta cell with two capillary contacts (shown with arrows). (**f**) Laminin is significantly enriched at both capillary interfaces compared with the lateral and apical regions. (**g**) Dlg is found across all of the cell membrane. (**h**) Par3 is significantly enriched in the apical domain compared with lateral or capillary regions. For all data, *n*=12 cells from 3 mice, 4 cells each; one-way ANOVA, Tukey’s multiple comparison tests and, as indicated on the graphs, *p<0.05, **p<0.01, ***p<0.001. Scale bars, 10 µm

We used immunofluorescence in mouse islets for laminin and E-cadherin, as in Fig. 1, and in addition stained for phosphorylated focal adhesion kinase (pFAK) which is a component of the integrin response (Fig. 2a). Fluorescence intensities showed, as expected, laminin enrichment at both capillary contacts and absence in the lateral domain (Fig. 2b,f). E-cadherin was enriched in the lateral domain, with some evidence for presence at the capillary interface (Fig. 2c). pFAK was enriched at both capillary interfaces, demonstrating an integrin response at each of the ECM contacts (Fig. 2d). We next studied the distribution of the polarity determinant proteins, discs large (Dlg) (basal) and partitioning defective protein 3 (Par3) (apical/junctional), in cells that contacted two capillaries (Fig. 2e). Dlg was present across all membranes (Fig. 2g) and Par3 was located away from both capillary interfaces, indicating enrichment in a putative apical domain (Fig. 2h).

**Fig. 3:**
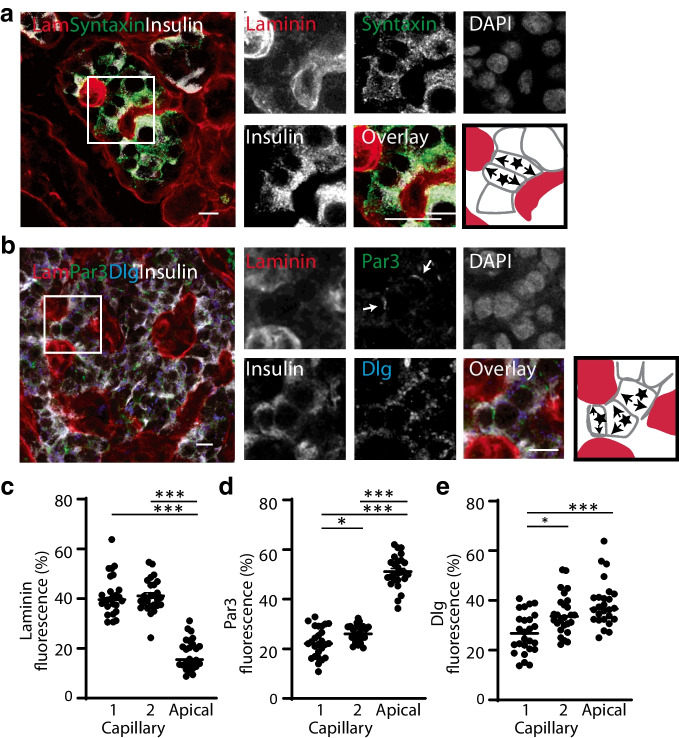
In human islets, regional enrichment of polarity determinants, Dlg and Par3, in cells contacting two capillaries. (**a**) Laminin (Lam) immunostaining identifies the capillaries, insulin the beta cells and syntaxin 1A the cell outlines. The cartoon identifies, with stars, two beta cells that contact two capillaries (arrows). (**b**) Immunostaining of Par3 shows enrichment in areas away from capillary contact points (arrows highlight the Par3 staining) with Dlg staining all around the cells, including the capillary contact points. The cartoon identifies, with stars, three beta cells that contact two capillaries (arrows). (**c**) Laminin is significantly enriched at both capillary interfaces compared with the apical region. (**d**) Par3 is significantly enriched in the apical domain compared with either capillary region. (**e**) Dlg is found across all of the cell membrane. For all data, *n*=5 islets, 29 cells and 3 donors; one-way ANOVA, Tukey’s multiple comparison tests and, as indicated on the graphs, *p<0.05, ***p<0.001. Scale bars, 10 µm

**Fig. 4:**
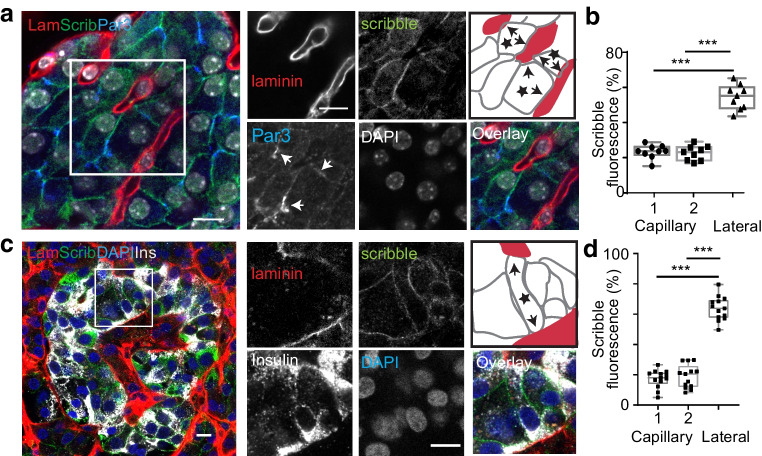
In mouse and human islets, regional localisation of the basolateral determinant scribble with cells that contact two capillaries. (**a**, **b**) In mouse islets, for beta cells that contact two capillaries, scribble is across all of the membrane but enriched at the lateral membrane. The cartoon identifies, with stars, three beta cells that contact two capillaries (arrows). Par3, the apical/junctional polarity determinant, is located away from the capillaries (arrows highlight the Par3 staining). Analysis of ROIs shows significant enrichment of scribble at the lateral membrane (*n*=9 cells; one-way ANOVA, Tukey’s multiple comparison tests, capillary 1/capillary 2 *p*=0.89 and, as indicated on the graphs, ***p<0.001). (**c**, **d**) In human islets, scribble shows a similar localisation to mouse and is present across all of the membrane, with enrichment at the lateral sides of the cells. The cartoon identifies, with a star, a beta cell that contacts two capillaries (arrows). Analysis of ROIs showed scribble was significantly enriched on the lateral surfaces (*n*=13 cells, 4 islets, 2 donors; one-way ANOVA, Tukey’s multiple comparison tests, capillary 1/capillary 2 *p*=0.86 and, as indicated on the graphs, ***p<0.001). Scale bars, 10 µm. Ins, insulin; Lam, laminin; Scrib, scribble

Immunostaining of human islets showed a similar organisation to mouse beta cells. In our hands the E-cadherin antibody gave poor staining in human islets, and therefore to outline the beta cells we stained for syntaxin 1A, which is enriched across all of the membrane (Fig. 3a) [10]. We used Par3 and Dlg immunostaining, which showed Par3 was enriched away from the capillaries and Dlg was present across all of the membrane (Fig. 3b–e). Further immunolabelling (Fig. 4) for the basolateral determinant scribble, which is a protein associated with cadherin junctions in epithelial cells, showed, in both mouse (Fig. 4a,b) and human (Fig. 4c,d) islets, that it was present across all of the beta cell membrane.

We conclude that in beta cells with two capillary contacts, a distinct form of polarisation develops with multiple basal domains.

### Both capillary contacts in beta cells are enriched in proteins specialised to regulate secretion

One functional consequence of ECM contact is to localise insulin secretion [29] through local activation of integrins [9] and local enhancement of the calcium response [11, 28]. We therefore tested whether both capillary contact points are specialised for secretion.

**Fig. 5:**
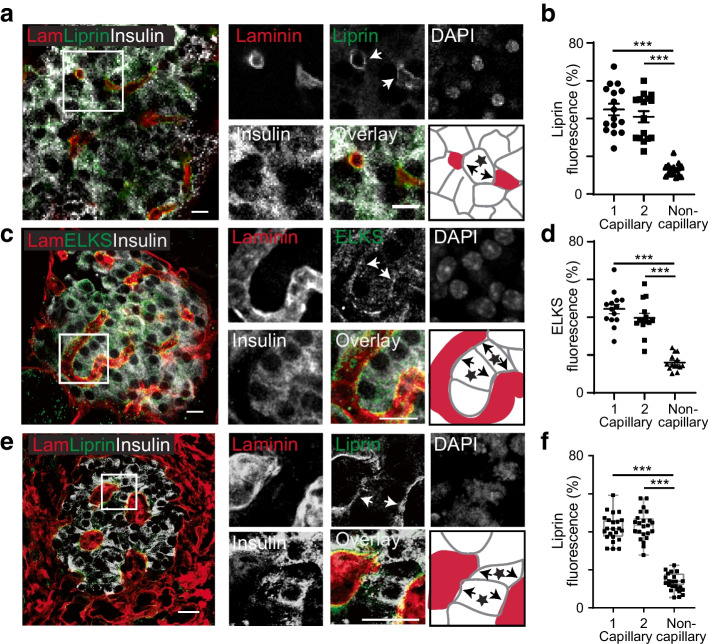
In mouse and human islets, beta cells that make two points of capillary contact show presynaptic scaffold proteins are enriched at both contact points. (**a**) In mouse islets, the immunostaining of laminin (Lam) identifies the capillaries and insulin the beta cells. Immunostaining of the presynaptic scaffold protein liprin α1 showed enrichment at both the capillary interfaces. The cartoon identifies, with a star, a beta cell with two capillary contacts. (**b**) Analysis of fluorescence intensity within ROIs shows significant liprin enrichment at the capillaries (*n*=16 cells, 4 islets, 3 mice; ANOVA, Tukey’s multiple comparisons, capillary 1/capillary 2 *p*=0.79 and, as indicated on the graphs, ***p<0.001). (**c**) In mouse islets, another presynaptic scaffold protein, ELKS, also showed enrichment at both the capillary interfaces. The cartoon identifies, with stars, two beta cells with two capillary contacts. (**d**) Analysis of fluorescence intensity within ROIs shows significant ELKS enrichment at the capillaries (*n*=14 cells, 5 islets, 5 mice; ANOVA, Tukey’s multiple comparisons, capillary 1/capillary 2 *p*=0.59 and, as indicated on the graphs, ***p<0.001). (**e**) In human islets we observed a similar enrichment of liprin α1 at the capillary interface. The cartoon identifies, with stars, two beta cells with two capillary contacts. (**f**) Analysis of fluorescence intensity within ROIs shows significant liprin enrichment at the capillaries (*n*=23 cells, 7 islets, 3 donors; ANOVA, Tukey’s multiple comparisons, capillary 1/capillary 2 *p*=0.59 and, as indicated on the graphs, ***p<0.001). Scale bars, 10 µm for the mouse islets and 20 µm for human

We used immunofluorescence for the presynaptic scaffold proteins, liprin and ELKS [30], which are important in controlling insulin secretion [11, 12]. In mouse islets, liprin (Fig. 5a,b) and ELKS (Fig. 5c,d) were significantly and equally enriched in beta cells at both capillary contacts. A similar enrichment of liprin was found at both capillary contact points in human beta cells (Fig. 5e,f). These results demonstrate that both capillary contacts in beta cells are enriched in synaptic scaffold proteins and potentially specialised for secretion.

### Both capillary contacts in beta cells are targets for insulin granule fusion

**Fig. 6:**
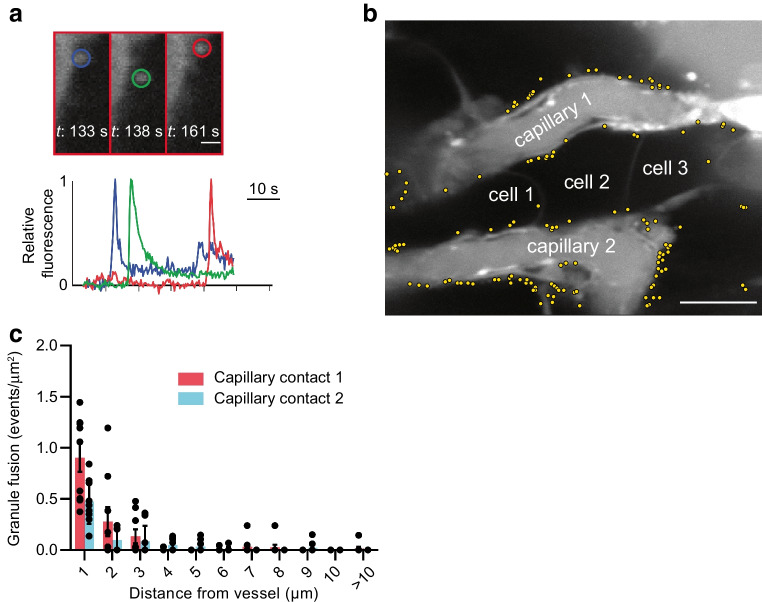
Glucose-dependent granule fusion is targeted to both points of capillary contact. (**a**) In live pancreatic slices, stimulated with high glucose (16.7 mmol/l) and forskolin (10 µmol/l), granule fusion events are transiently labelled by the sudden influx of extracellular dye, SRB, into the granule. In this example image, three granule fusion events (identified by coloured circles) occurred at different times but within proximity, and plotting the fluorescence changes over time shows the characteristic peak and decay of fluorescence as the granules fuse. Scale bar, 1 µm. (**b**) Measured over 20 min of high glucose and forskolin stimulation, the location of each identified granule fusion event is shown as a yellow dot. The distribution of granule fusion events in cells that contact two capillaries (capillary 1 and capillary 2, identified by the extracellular dye) is strongly enriched at both contact points. Scale bar, 10 μm. (**c**) The frequency of granule fusion events in each cell, per length of capillary, was significantly clustered towards the capillaries (Wilcoxon matched pairs signed rank test comparing <1 µm away with >1 µm away, *p*<0.05). However, the number of fusion events at, or close to, one capillary was the same as the other capillary (*n*=9 cells, 5 islets and 3 mice; Wilcoxon matched pairs signed rank test, *p*=0.10). *t*, time

To identify the significance on secretion, we used live-cell experiments with an extracellular fluid-phase marker to record granule fusion events. Each individual fusion event is observed as a sudden increase in fluorescence as the extracellular dye enters the fusing granule, which decays over time as the granule collapses into the cell membrane (see Fig. 6a) [31]. We have shown this fluorescence signature co-localises with immunostained insulin and overlaps with the efflux of expressed proinsulin-GFP [32], giving us an indirect method for determining insulin granule fusion events in space and time [10, 28, 31].

Mouse pancreatic slices were stimulated for 20 min with 16.7 mmol/l glucose plus 10 µmol/l forskolin (Fig. 6b). Each individual fusion event is marked with a yellow dot, enabling us to map out the cumulative spatial pattern of granule fusion over the 20 min of stimulation (Fig. 6b). In this example, three cells each contact two capillaries (Fig. 6b). Analysis shows that both capillary contacts are the target for granule fusion (Fig. 6b,c).

We conclude that in beta cells with two capillary contacts, both contacts support exocytosis and are functionally equivalent for secretion.

### Granule fusion at the capillary contacts can be differentially controlled

Past work on beta cells with one point of capillary contact shows this contact is the target for granule fusion [28], local enhancement of the calcium response [11, 28] and enrichment of synaptic scaffold proteins [10]. This has led to the proposal that the capillary interface of beta cells has a presynaptic-like organisation that locally regulates secretion [33]. If this is the case, then the two points of contact might function somewhat independently of each other due to locally different regulation or structures.

**Fig. 7:**
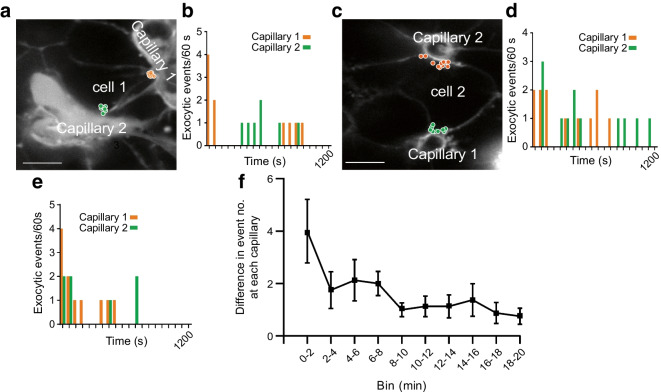
Exocytic event timing is different at each capillary contact point. Granule fusion events induced by high glucose (16.7 mmol/l) and forskolin (10 µmol/l) for 20 min were identified. (**a**) Exocytic events in one cell contacting two capillaries are identified in space by the coloured circles. (**b**) The same data as in (**a**) showing the timing of each fusion event (in 60 s bins) during glucose stimulation. Analysis of this example showed no significant correlation between the occurrences of fusion events in one capillary vs the other (Spearman’s correlation analysis *r*_*s*_=−0.22, *p*=0.35). (**c**, **d**) Another example response to glucose. Analysis again showed no significant correlation between the occurrences of fusion events in one capillary vs the other (Spearman’s correlation analysis *r*_*s*_=−0.09, *p*=0.69). (**e**) An example where the occurrence of fusion events in one capillary was correlated with the other (Spearman’s correlation analysis *r*_*s*_=0.51, *p*<0.05). (**f**) Plots of the difference in fusion event numbers between each capillary contact, per time bin, show the biggest difference at the beginning of glucose stimulation (*n*=9 cells, 5 islets, 3 mice). Scale bars, 5 µm

To test this, mouse pancreatic slices were stimulated with glucose (16.7 mmol/l) and forskolin (10 µmol/l) for 20 min and exocytic events recorded as before in space (Fig. 7a,c) and now in time over the period of stimulation (Fig. 7b,d). Interestingly, the temporal patterns of granule fusion events per 60 s time bin were different for each of the capillary contact regions. When this difference was plotted out over time, it appeared that the biggest difference occurred at the earlier time points (Fig. 7f). A Spearman correlation analysis of the occurrence of fusion events at each contact in each time bin showed no significant correlation in 10/12 cells. The two cells with significant correlations (*p*<0.05) both had a positive correlation (*r*_*s*_=0.51 and 0.55), indicating fusion events at each contact point were coordinated (an example cell is shown in Fig. 7e). These data demonstrate that granule exocytosis at each capillary contact can be differentially controlled, providing further evidence for very local, presynaptic-like control of granule fusion.

### Greater capillary contact area is linked to more insulin secretion

**Fig. 8:**
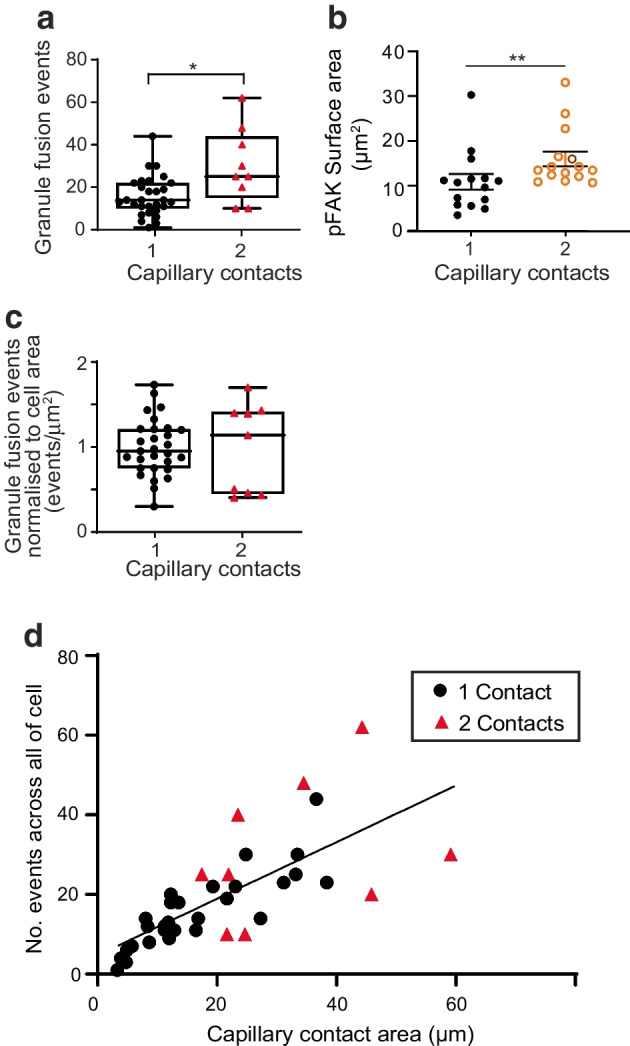
Beta cells with two capillary contacts have more insulin granule fusion events. (**a**) The total number of granule fusion events per cell in response to 16.7 mmol/l glucose and 10 µmol/l forskolin for 20 min was significantly greater for cells making contact with two capillaries than for cells making contact with one capillary (*n*=38 cells, 5 islets, 3 mice; Mann–Whitney test, *p<0.05). (**b**) The pFAK surface area was significantly larger in beta cells making contact with two capillaries than in cells making contact with one capillary (*n*=15 cells, 3 islets, 3 mice; Mann–Whitney test, **p<0.01). (**c**) The granule fusion data now normalised for cell surface area show no difference in the number of granule fusion events per µm^2^ (*n*=38 cells, 5 islets, 3 mice; Mann–Whitney test, *p*=0.92). (**d**) A plot of total granule fusion events per cell plotted against total capillary area per cell approximated to a linear relationship, with cells contacting one capillary (circles) intermixed with cells contacting two capillaries (red triangles) (*n*=38 cells, 5 islets, 3 mice; line fitted to all data points with a slope of 0.71, *R*^2^ 0.52, significant, *p*<0.001)

We next compared secretion from beta cells with one capillary contact with secretion from those with two. We reanalysed our data to determine the total number of granule fusion events in each cell (note, this is the number of events per cell within the optical slice). The results show significantly more granule fusion events in the cells with two contacts, compared with cells with one (Fig. 8a). However, the surface area of contact with capillaries, as measured by pFAK staining area, was also significantly greater for cells with two contacts (Fig. 8b). When normalised against contact area there was no difference in granule fusion density between cells with one or two capillary contacts (Fig. 8c), suggesting that the underlying mechanisms that control granule fusion are similar in both cell populations. Furthermore, the plot, for each cell, of the total area of capillary contact, against the total number of granule fusion events, is linearly related (linear regression, *p*<0.001), with overlap in the distributions for cells with one or two capillary contacts (Fig. 8d).

These findings lead to an interesting possibility that overall islet secretory output is determined by the sum of the total contact area of all the beta cells with the capillaries.

### Type two diabetes and changes in capillary structure

**Fig. 9:**
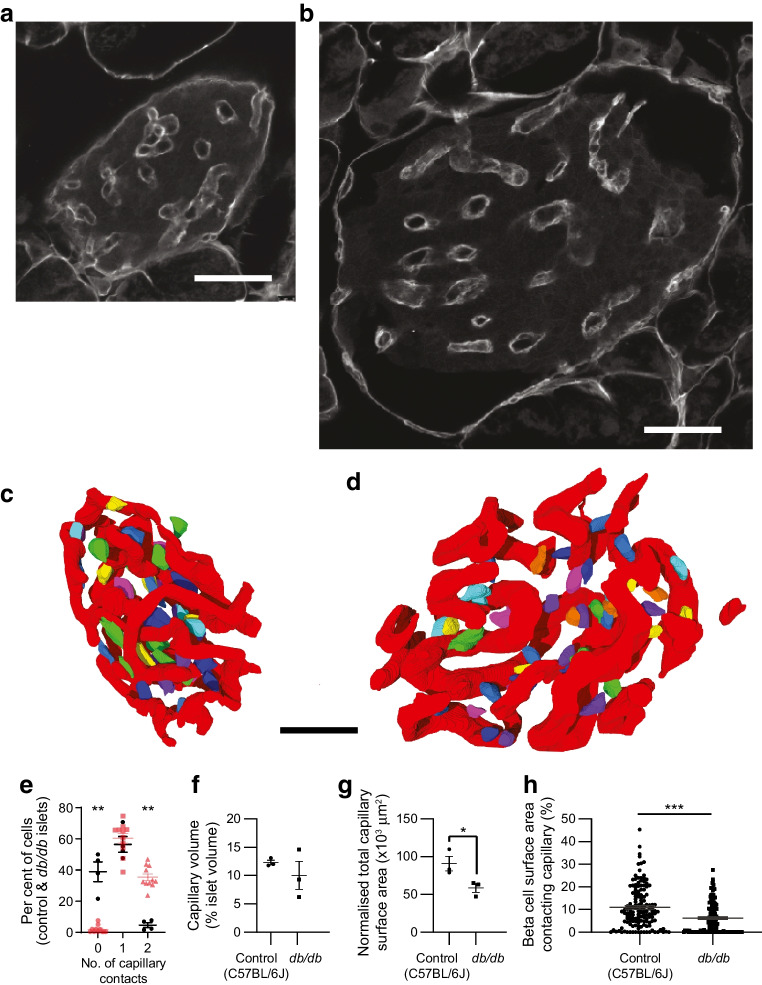
Capillary structure changes in the *db*/*db* mouse model of diabetes impact on beta cell capillary contacts. (**a**) A control islet immunostained with laminin and (**b**) an islet from a *db*/*db* mouse stained with laminin show enlargement of the islet and changes to capillary structure. (**c**) Control and (**d**) *db*/*db* islets show segmentation of confocal image stacks outlining the 3D capillary structure (red) and the relationship with a randomly selected set of beta cells (multiple colours) within the islet. (**e**) The proportion of cells contacting zero, one or two capillaries was estimated using serial sectioning (as in Fig. 1, *n*=4 *db*/*db* islets from 3 mice). For comparison, the red symbols are the same data as in Fig. 1b from C57 control mice. Mann–Whitney tests showed significant differences between control and *db*/*db* islets in the numbers with zero (**p<0.01) and two (**p<0.01) contacts but not with one contact (*p*=0.49). (**f**) Analysis of the segmented images showed no difference in total capillary volume expressed as a proportion of total islet volume in *db*/*db* islets compared with control islets (*n*=3 control islets and 3 *db*/*db* islets, each from 3 mice; two-sided Student’s *t* test, *p*=0.41). (**g**) However, capillary complexity was reduced in *db*/*db* islets and this was identified as a decrease in the total capillary surface area, normalised to total islet volume (*n*=3 control islets and 3 *db*/*db* islets, each from 3 mice; two-sided Student’s *t* test, *p<0.05). (**h**) The capillary contact area per beta cell was reduced in *db*/*db* islets (*n*=294 cells, 6 islets, 6 mice; two-sided Student’s *t* test, ***p<0.001). Scale bars, 50 µm

In terms of disease, islet capillary structure changes in type two diabetes, described as a decrease in capillaries in rodents [19, 23] or an increase in humans [20]. In the light of our findings, we wanted to determine whether changes in capillary structure affect the beta cell contacts using the *db*/*db* mouse model of type 2 diabetes. To analyse the 3D data, we segmented the images to define the capillaries (with laminin) and a subset of beta cells (with insulin and E-cadherin). We found changes in capillary shape in disease, as shown with the immunostaining (Fig. 9a,b) and in the segmented images (Fig. 9c,d). Determination of the proportion of cells that make one or two capillary contacts, using serial immunofluorescence images, showed an almost complete loss of cells with two capillary contacts (Fig. 9e) and an increase in cells with no apparent contacts (compare Fig. 1b with Fig. 9e). Relative to islet size, analyses of the segmented data showed that the volume of capillaries does not change (Fig. 9f); however, there was a significant decrease in the capillary surface area (Fig. 9g). Analysis of the segmented data for beta cell contacts showed that this change in capillary structure reduced the overall contact area of each beta cell with the capillary (Fig. 9h).

We conclude that the diabetes-induced changes in islet capillary structure do impact the relationship with beta cells and decrease the beta cell contact area with the capillary ECM.

## Discussion

We identify that 36% of mouse and 22% of human beta cells contact two capillaries. These cells have an unusual form of cell polarity, with a basal domain at each contact and an apical domain away from the capillaries (Figs 2, 3 and 4). Each capillary contact is enriched in synaptic scaffold proteins and is a target for insulin granule fusion (Figs 5, 6 and 7). Intriguingly, the contact points can function independently, indicating very local control of secretion (Fig. 7). Independent of the number of capillary contacts made by a beta cell, increased insulin secretion is correlated with increased capillary contact area (Fig. 8d).

In the *db*/*db* mouse model of diabetes, we observe that the total capillary volume per islet is unchanged, but there is a significant reduction both in capillary surface area per islet and in the area of capillary contact per individual beta cell, and an almost complete loss of cells with two capillary contacts (Fig. 9). These changes could contribute to the reduced secretion in disease and to disease progression.

These insights identify the beta cell to capillary contacts as a new factor defining beta cell polarity, regulating insulin secretion and undergoing significant changes in disease.

### Is this a distinct subpopulation of beta cells?

Heterogeneity across beta cells within an islet includes mature vs immature beta cells [34], functional distinctions that lead to distinct cell responses [35] and markers for different cell subtypes [36]. We have segregated beta cells into two populations based on an anatomical distinction and show an impact on cell polarity and insulin secretion. In this way, the beta cells with two capillary contacts are distinct: they have a different form of polarity and the larger area of contact with capillaries drives bigger responses to glucose.

### The beta cell capillary interface as a key factor in controlling insulin secretion

Our data show most beta cells contact two obviously separate capillaries, but we cannot exclude that sometimes the same capillary makes both contacts. This could be important if populations of capillaries of the islet were segregated into distinct functions and reminds us of arguments around whether there is a systematic blood flow through the islet (e.g. core to periphery) [37]. The most recent data show no specialised flow [18, 38] and instead indicate an interleaving of islet capillaries with the surrounding acinar tissue [18]. This does not exclude the idea of specialised capillaries but is consistent with the similar function we show for both capillary contacts.

Our conclusion that beta cell capillary contact area is a key driver for insulin secretion is new but is consistent with past work. ECM proteins enhance beta cell proliferation and secretion [29, 39] and one route for these effects is through integrin and focal adhesion kinase (FAK) activation [9, 28, 29]. What is new here is that the contact area for each cell appears to be a limiting factor for insulin secretion: the greater the contact area, the greater the secretion. It should be noted that the data that support this idea (Fig. 8) are analyses of two-photon data (Fig. 6). These experiments count the number of exocytic events just in that optical plane and not across the whole cell. However, the correlation we see (Fig. 8d) and the data overlap between cells with one and two capillary contacts (Fig. 8c) is strong evidence that contact area alone is a limiting step in granule fusion.

The limiting factor(s) could be structural, such as limited access of granules to the cell membrane, or a signalling factor, such as localised calcium entry. It is intriguing that we now show that the two contact points have evidence for independent function. This suggests local control, at the contact points, of key elements of the stimulus–secretion cascade. This idea is supported by work showing local enhanced calcium responses at the capillary interface [11, 28] and that sites of insulin granule fusion are enriched in proteins that control exocytosis [40].

### Broader relevance of our findings

Our work uses pancreatic slices, where islet structure is well preserved, and, by comparison with isolated islets, slices have fast calcium waves and enhanced glucose-dependent insulin secretion [28]. The slice therefore represents an excellent in vitro system. However, both external innervation and blood flow are lost in slices and both these factors might influence beta cell biology [41]. Notwithstanding these drawbacks, the slice is an important tool for understanding islet biology and is a considerable advance on the use of isolated islets.

It is important to note that we focus on the response of beta cells to the capillary ECM [15]. However, beta cell function is also influenced by other interactions with capillary cells, including endothelial cells [21, 42] and pericytes, which affect beta cell maturation and function [43] and alter islet blood flow [44] to regulate insulin secretion [45].

### Impact of islet vascular changes in type 2 diabetes

A defining characteristic of type 2 diabetes is changes to the systemic capillary network [46, 47]. In islets, changes to capillaries have been observed but are difficult to define [20–22, 48] because of the complexity of the volumes and changes in the overall size and structure of the islet which alter over the progression of the disease [24, 49]. However, our work clearly identifies a common factor in diseased islets. The islets we studied were from mice with overt diabetes where capillaries got bigger with less complexity, measured in terms of a reduction in capillary surface area per islet volume. This result is reinforced with measurements on single cells which show a significant reduction in percentage area of capillary contact (Fig. 9).

We speculate that our findings identify a novel vicious cycle that propels disease progression. In early disease, changes in cell signalling and compensatory increases in islet beta cell mass [49] might be paralleled with changes in capillaries to preserve beta cell capillary contact area and secretory output. However, as disease progresses hyperglycaemia damages capillary structure which decreases beta cell capillary contact area and leads to a consequent reduction in insulin secretion, further accelerating loss of glucose homeostasis. This might be compounded by the glucotoxic damage and loss of beta cells in diabetes which would be expected to reduce vascular endothelial growth factor A (VEGFA) secretion from beta cells [50, 51], which is a known driver of islet vascularisation.

### Supplementary Information

Below is the link to the electronic supplementary material.

ESM Table (PDF 131 KB)

## Data Availability

All data generated or analysed during this study are included in the article. No additional resources were generated or analysed during the current study.

## Abbreviations

Dlg: Discs large
E-cadherin: Epithelial cadherin
ECM: Extracellular matrix
KRBH: Krebs Ringer bicarbonate buffer
Par3: Partitioning defective protein 3
pFAK: Phosphorylated focal adhesion kinase
ROI: Region of interest
SRB: Sulforhodamine B
