# Supplementary material for: Capillary contact points determine beta cell polarity, control secretion and are disrupted in the db/db mouse model of diabetes
Source: Diabetologia. 2024 May 30;67(8):1683–97. doi: 10.1007/s00125-024-06180-x (PMC11343897; doi:10.1007/s00125-024-06180-x)
Supplement: Supplementary file 1 — ESM Table (PDF 131 KB) [file 125_2024_6180_MOESM1_ESM.pdf]

Electronic Supplementary Material (ESM)

Jevon et al
Capillary contact points determine beta cell polarity, control secretion and are disrupted in the *db/db* mouse model of diabetes
ESM Tables

ESM Table 1: Characteristics of human pancreatic tissue donors

Sample ID	Age	Sex	Source	Pancreatectomy or donor	Diabetes (Y/N), type and duration	Figure
Sample 1	49	F	Royal North Shore Hospital	Partial Pancreatectomy	N	1,3,5
Sample 2	64	F	Royal North Shore Hospital	Partial Pancreatectomy	N	4,5
Sample 3	73	M	Royal North Shore Hospital	Partial pancreatectomy	N	3
Sample 4	68	F	St Vincent's Institute	Cadaveric donor	N	1,3,5
Sample 5	37	M	St Vincent's Institute	Cadaveric donor	N	4
